# Supplementary material for: A cytoplasmic peptidoglycan amidase homologue controls mycobacterial cell wall synthesis
Source: eLife. 2016 Jun 15;5:e14590. doi: 10.7554/eLife.14590 (PMC4946905; doi:10.7554/eLife.14590)
Supplement: Supplementary file 1. — (a) Strains. Strains used, with strain number, abbreviated name used in the text and full genotype. (b) Plasmids. Plasmids used, with original reference for parent plasmid, and strain cross-reference. (c) Primers. Primers used to construct each strain. Those beginning with ‘p’ are plasmids, others are recombineered alterations on the chromosome. (d) Kinetic parameters of MurA proteins pulled from the literature. DOI: http://dx.doi.org/10.7554/eLife.14590.016 [file elife-14590-supp1.docx]

Supplementary file 1

| **Supplementary file 1A. Strains** | | | |
| --- | --- | --- | --- |
| Strain # | Nickname | Genotype | Used in Figures |
| CB001 | *Msmeg* | *Mycobacterium smegmatis* mc^2^155 | 6A, S2B |
| CB082 | Ptet::*cwlM* | mc^2^155 ∆*cwlM*::zeoR L5::pL5tetOR-*cwlM* / petetR | 2BDEF |
| CB100 | *cwlM*::FLAG | mc^2^155 *cwlM*::FLAG-zeoR | 2AB, 6B, S2C |
| CB193 | *cwlM* OE | mc^2^155 / pUVtetOR-*cwlM*-his | S1 |
| CB194 | *cwlM* E209A D331A OE | mc^2^155 / pUVtetOR-*cwlM* E209A D331A-his | S1 |
| CB202 | *cwlM*_amidase_ OE | mc^2^155 / pUVtetOR-*cwlM*_amidase domain_-his | S1 |
| CB233 |  | mc^2^155 ∆*cwlM*::zeoR L5::pL5tetOR-*cwlM* | Parent |
| CB236 | L5::*cwlM* | mc^2^155 ∆*cwlM*::zeoR L5:: KK216-*cwlM* | 1G, S2B |
| CB239 | L5::*cwlM* E209A D331A | mc^2^155 ∆*cwlM*::zeoR L5:: KK216-*cwlM* E209A D331A | 1G |
| CB265 |  | mc^2^155 ∆CwlM::zeoR L5-KK216-CwlM T35A | S2A |
| CB300 | L5::*cwlM­*-FLAG | mc^2^155 ∆*cwlM*::zeoR L5::KK216-*cwlM*-FLAG | 2CEF, S2AB |
| CB319 | L5::*cwlM* T374A-FLAG | mc^2^155 ∆ *cwlM* ::zeoR L5::KK216-*cwlM* T374A-FLAG | 2CEF, S2A, S4 |
| CB345 | L5::*cwlM* T376A-FLAG | mc^2^155 ∆ *cwlM* ::zeoR L5::KK216-*cwlM* T376A-FLAG | 2CEF, S2A |
| CB348 | L5::*cwlM* T378A-FLAG | mc^2^155 ∆ *cwlM*::zeoR L5::KK216-*cwlM* T378A-FLAG | 2CEF, S2A |
| CB351 | L5::*cwlM* K362A-FLAG | mc^2^155 ∆*cwlM*::zeoR L5::KK216-*cwlM* K362A-FLAG | S2A |
| CB354 | L5::*cwlM* K369A-FLAG | mc^2^155 ∆*cwlM*::zeoR L5::KK216-*cwlM* K369A-FLAG | S2A |
| CB418 | *cwlM*::FLAG / PknB OE | mc^2^155 *cwlM*::FLAG-zeoR / pUV15tetOR-PknB(TB) | 3B |
| CB419 |  | ArcticExpress RP DE3 / pHGWA – CwlM_TB_ | 3A, 5, S3BC, S5 |
| CB457 | L5::*cwlM*1cys-strep | mc^2^155 ∆*cwlM*::zeoR L5::KK216-*cwlM* C144S C310S-strep | 2G |
| CB663 | L5::*cwlM*-strep | mc^2^155 ∆*cwlM*::zeoR L5::KK216-*cwlM*-strep | 2D, S2C |
| CB666 | L5::*cwlM* T374A-strep | mc^2^155 ∆*cwlM*::zeoR L5::KK216-*cwlM* T374A-strep | 2D |
| CB669 | L5::*cwlM* T376A-strep | mc^2^155 ∆*cwlM*::zeoR L5::KK216-*cwlM* T376A-strep | 2D |
| CB672 | L5::*cwlM* T378A-strep | mc^2^155 ∆*cwlM*::zeoR L5::KK216-*cwlM* T378A-strep | 2D |
| CB675 | L5::*cwlM* K362A K369A T376A T378A-strep | mc^2^155 ∆CwlM::zeoR L5::KK216-CwlM K362A K369A T376A T378A-strep | S2C |
| CB706 | *lprG*-strepC | mc^2^155 L5::KK216-*lprG*-strepC |  |
| CB737 | L5::*cwlM*(nuoR) tw::*murA* | mc^2^155 ∆*cwlM*::lox ∆*murA*1::lox ∆*murA2*::lox L5::KK158 -P*_cwlM_*-*cwlM* tw::pDE43MCtZ -P*murA*-*murA*-strep | 4BC |
| CB757 |  | ArcticExpress RP DE3 / pET28 – his-MurA_TB_ | 5, S5 |
| CB762 | L5::*cwlM*(nuoR) tw::*murA* S368P | mc^2^155 ∆*cwlM*::lox ∆*murA*1::lox ∆*murA2*::lox L5::KK158 -P*cwlM*-*cwlM* tw::pDE43MCtZ –P*murA*-*murA* S368P-strep | 4B |
| CB779 | *murA* WT | mc^2^155 ∆*cwlM*::lox ∆*murA*1::lox ∆*murA*2::lox L5::KK216-*cwlM*-FLAG tw::pDE43MCtZ - P*murA*-*murA*-strep | 4BC, 6, S6 |
| CB782 | *murA* S368P | mc^2^155 ∆*cwlM*::lox ∆*murA*1::lox ∆*murA*2::lox L5::KK216-*cwlM*-FLAG tw::pDE43MCtZ - P*murA*-*murA* S368P-strep | 4B, 6, S6 |
| CB785 | *cwlM* T374A *murA* S368P | mc^2^155 ∆*cwlM*::lox L5::KK216-*cwlM* T374A-FLAG tw::pDE43MCtZ - P*murA*-*murA* S368P-strep ∆*murA*1::lox ∆*murA*2::lox | 4B, S4 |
| CB884 |  | ArcticExpress RP DE3 / pET28 – his-MurA_MS_ | S5B |
| CB885 |  | ArcticExpress RP DE3 / pET28 – his-MurA_MS_S368P | S5B |
| CB886 | *Pgm*-strepC | mc^2^155 L5::KK216 - pgm-strepC | 2G |
| CB899 |  | ArcticExpress RP DE3 / pHGWA – his-CwlM_TB_ T382A | 5F |
| CB900 |  | ArcticExpress RP DE3 / pHGWA – his-CwlM_TB_ T382A T384A T386A | 5F |

| **Supplementary file 1B. Plasmids**  * indicates that the citation is for the parent plasmid, which was altered for this study | | | |
| --- | --- | --- | --- |
| Strain # | Plasmid name | Used in: | Reference |
| CB018 | pL5tetOR-*cwlM* | CB82, CB233 | *(Ehrt, 2005) |
| CB028 | pCreSacBR (kan) | CB737, CB762 | Gift of Adrie Steyn |
| CB081 | petetR | CB82 | (Ehrt, 2005) |
| CB181 | pKK216-*cwlM* | CB236 | *(Kieser et al., 2015) |
| CB182 | pKK216-*cwlM* E209A D331A | CB239, CB737 | *(Kieser et al., 2015) |
| CB193 | pEtetOR-*cwlM*-his | CB193 | *(Ehrt, 2005) |
| CB194 | pEtetOR-*cwlM* E209A D331A-his | CB194 | *(Ehrt, 2005) |
| CB202 | pEtetOR-*cwlM*_amidase domain_-his | CB202 | *(Ehrt, 2005) |
| CB254 | pKK216-*cwlM* T35A-FLAG | CB265 | *(Kieser et al., 2015) |
| CB255 | pKK216-*cwlM* T374A-FLAG | CB319, CB785 | *(Kieser et al., 2015) |
| CB277 | pKK216-*cwlM*-FLAG | CB300, CB779, CB782 | *(Kieser et al., 2015) |
| CB340 | pKK216-*cwlM* T376A-FLAG | CB345 | *(Kieser et al., 2015) |
| CB341 | pKK216-*cwlM* T378A-FLAG | CB348 | *(Kieser et al., 2015) |
| CB342 | pKK216-*cwlM* K362A-FLAG | CB351 | *(Kieser et al., 2015) |
| CB343 | pKK216-*cwlM* K369A-FLAG | CB354 | *(Ehrt, 2005; Kieser et al., 2015) |
| CB411 | pUVtetOR-PknB_TB_ | CB418 | (Ehrt, 2005; Gee et al., 2012) |
| CB419 | pHGWA-his-*cwlM*_TB_ | CB419 | *Accession: [EU680840](http://getentry.ddbj.nig.ac.jp/getentry/na/EU680840/?filetype=html) |
| CB452 | pKK216-*cwlM* C144S C310S-strep | CB457 | *(Kieser et al., 2015) |
| CB557 | pKK216-P_cwlM_-FLAG-*cwlM*∆CT | Fig. 4B | *(Kieser et al., 2015) |
| CB558 | pKK216-mRFP-strep | Fig. 4B | *(Ehrt, 2005; Kieser et al., 2015) |
| CB634 | pKK158-P_cwlM_-*cwlM* | CB737 | *(Ehrt, 2005; Kieser et al., 2015) |
| CB658 | pKK216-*cwlM*-strep | CB663 | *(Ehrt, 2005; Kieser et al., 2015) |
| CB659 | pKK216-*cwlM* T374A-strep | CB666 | *(Kieser et al., 2015) |
| CB660 | pKK216-*cwlM* T376A-strep | CB669 | *(Kieser et al., 2015) |
| CB661 | pKK216-*cwlM* T378A-strep | CB672 | *(Kieser et al., 2015) |
| CB662 | pKK216-*cwlM* K362A K369AT376A T378A-strep | CB675 | *(Kieser et al., 2015) |
| CB681 | pDE43MCtH-P_MurA_-*murA* S368P-strep | CB737 | *(Blumenthal et al., 2010; Kieser et al., 2015) |
| CB703 | pKK216-*lprG*-strepC | CB706 | *(Kieser et al., 2015) |
| CB736 | pDE43MCtZ-P_MurA_-*murA*-strep | CB737 | *(Blumenthal et al., 2010) |
| CB748 | pDE43MCtZ-P_murA_-*murA* S368P-strep | CB762 | *(Blumenthal et al., 2010) |
| CB757 | pET28-his-­*murA*_TB_ | CB757 | *https://www.addgene.org/vector-database/2565/ |
| CB882 | pKK216-*pgm*-strepC | CB886 | *(Kieser et al., 2015) |
| CB884 | pET28-his-*murA*_MS_ | CB884 | *https://www.addgene.org/vector-database/2565/ |
| CB885 | pET28-his-*murA*_MS_S3868P | CB885 | *https://www.addgene.org/vector-database/2565/ |
| CT104 | pNit-RecET-SacBR (gent) | CB82,CB233,CB737 | (van Kessel and Hatfull, 2006) |
| CB897 | pHGWA-his-*cwlM*_TB_ T382A | CB899 | *Accession: [EU680840](http://getentry.ddbj.nig.ac.jp/getentry/na/EU680840/?filetype=html) |
| CB898 | pHGWA-his-*cwlM*_TB_ T382A T384A T386A | CB900 | *Accession: [EU680840](http://getentry.ddbj.nig.ac.jp/getentry/na/EU680840/?filetype=html) |

| **Supplementary file 1C. Primers** | | |
| --- | --- | --- |
| Strain # | feature | primers |
| CB082 | ∆*cwlM*::zeoR | ATATGGATCCgacgattcgttctccacc  ATTATACGAAGTTATggactagctaggcagca  tgctgcctagctagtccATAACTTCGTATAAT  aacgtgaaagtgcctgtATAACTTCGTATAGC  GCTATACGAAGTTATacaggcactttcacgtt  GATCGGTACCgaggcattcgcgtaca |
|  | pL5tetOR-*cwlM*  (also used in CB233) | ATATTTAATTAAtcaaccagcctggagg  ATATGAATtcaggcgccgcggccgg |
| CB100 | *cwlM*::FLAG-zeoR | ATATGATATccccacgggcccatca  Aggttctcactaccggcgccgcggccgg  Ccggccgcggcgccggtagtgagaacctg  gttacgtggaagggcGCTCGAGATAACTTCG  GAAGTTATCTCGAGCgcccttccacgtaac  ATATGATATCcgacgcgatcctgctga |
| CB193 | pUVtetOR-*cwlM*-his | ATATTTAATTAAtcaaccagcctggagg  TGGTGATGGTGATGACAggcgccgcggccggc  ATATGAATTCtcaGTGATGGTGATGGTGATGA |
| CB194 | pUVtetOR-*cwlM* E209A D331A-his | ATATTTAATTAAtcaaccagcctggagg  TGGTGATGGTGATGACAggcgccgcggccggc  ATATGAATTCtcaGTGATGGTGATGGTGATGA  Caggatgtcggccgcactgatgggccc  gggcccatcagt gcg gccgacatcctg  gatgtagccgatggcgacctgcacggt  accgtgcaggtcgccatcggctacatc |
| CB202 | pUVtetOR-*cwlM*_amidase domain_-his | TTAATTAAtcaaccagcctggaggggccccgt  Gccggtgacgcgtgacgctgccagtgctgc  gca gca ctg gca gcg tca cgc gtc acc ggc  TGGTGATGGTGATGACAggcgccgcggccggc  ATATGAATTCtcaGTGATGGTGATGGTGATGA |
| CB236 | pKK216-*cwlM* | ATATTTAATTAAtcaaccagcctggagg  TGGTGATGGTGATGACAggcgccgcggccggc  ATATGAATTCtcaGTGATGGTGATGGTGATGA |
| CB239 | pKK216-*cwlM* E209A D331A | ATATTTAATTAAtcaaccagcctggagg  TGGTGATGGTGATGACAggcgccgcggccggc  ATATGAATTCtcaGTGATGGTGATGGTGATGA  Caggatgtcggccgcactgatgggccc  gggcccatcagt gcg gccgacatcctg  gatgtagccgatggcgacctgcacggt  accgtgcaggtc gcc atcggctacatc |
| CB265 | pKK216-CwlM T35A | ATATTTAATTAAtcaaccagcctggagg  ATATCATATCcgacgcgatcctgctga  Cgccacgtggcggcccgcgctcaggtcagtgtc  gac act gac ctg agc gcg ggc cgc cac gtg gcg |
| CB300 | pKK216-*cwlM*-FLAG | ATATTTAATTAAtcaaccagcctggagg  ATATCATATCcgacgcgatcctgctga |
| CB319 | pKK216-*cwlM* T374A-FLAG | ATATTTAATTAAtcaaccagcctggagg  ATATCATATCcgacgcgatcctgctga  Gaacgtgaaagtgcctgcggggcggtcgttctt  aag aac gac cgc ccc gca ggc act ttc acg ttc |
| CB345 | pKK216-*cwlM* T376A-FLAG | ATATTTAATTAAtcaaccagcctggagg  ATATCATATCcgacgcgatcctgctga  Ttcggcgaacgtgaaagcgcctgtggggcggtcgtt  aacgaccgccccacaggcgctttcacgttcgccgaa |
| CB348 | pKK216-*cwlM* T378A-FLAG | ATATTTAATTAAtcaaccagcctggagg  ATATCATATCcgacgcgatcctgctga  Gagcagttcggcgaacgcgaaagtgcctgtggg  ccc aca ggc act ttc gcg ttc gcc gaa ctg ctc |
| CB351 | pKK216-*cwlM* K362A-FLAG | ATATTTAATTAAtcaaccagcctggagg  ATATCATATCcgacgcgatcctgctga  Cagcaggtacaggcgcgccacggccgcgagaac  gttctcgcggccgtggcgcgcctgtacctgctg |
| CB354 | pKK216-*cwlM* K369A-FLAG | ATATTTAATTAAtcaaccagcctggagg  ATATCATATCcgacgcgatcctgctga  Tgtggggcggtcgttcgcgcccagcaggtacag  ctgtacctgctgggcgcgaacgaccgccccaca |
| CB419 | pHGWA-his-*cwlM*_TB_ | Ggggacaagtttgtacaaaaaagcaggcttcatgccgagtccgcgccgcgaa  GGGGACCACTTTGTACAAGAAAGCTGGGTGtcaagaaccgccgagtctacccg |
| CB457 | pKK216-*cwlM* C144S C310S-strep | ATATTTAATTAAtcaaccagcctggagg  TATATATAAAGCTTTCACTTCTCGAACTGGGGGT  Cagggtctccgggccggagatcccgtcgggata  tatcccgacgggatctccggcccggagaccctg  gcggccgtgtgtgcgggagtcacgtaaaccggt  accggtttacgtgactcccgcacacacggccgc |
| CB663 | pKK216-*cwlM*-strep | ATATTTAATTAAtcaaccagcctggagg  TATATATAAAGCTTTCACTTCTCGAACTGGGGGT |
| CB666 | pKK216-*cwlM* T374A-strep | ATATTTAATTAAtcaaccagcctggagg  TATATATAAAGCTTTCACTTCTCGAACTGGGGGT  Gaacgtgaaagtgcctgcggggcggtcgttctt  aagaacgaccgccccgcaggcactttcacgttc |
| CB669 | pKK216-*cwlM* T376A-strep | ATATTTAATTAAtcaaccagcctggagg  TATATATAAAGCTTTCACTTCTCGAACTGGGGGT  Ttcggcgaacgtgaaagcgcctgtggggcggtcgtt  aacgaccgccccacaggcgctttcacgttcgccgaa |
| CB672 | pKK216-*cwlM* T378A-strep | ATATTTAATTAAtcaaccagcctggagg  TATATATAAAGCTTTCACTTCTCGAACTGGGGGT  Gagcagttcggcgaacgcgaaagtgcctgtggg  cccacaggcactttcgcgttcgccgaactgctc |
| CB675 | pKK216-CwlM K362A K369A T376A T378A-strep | ATATTTAATTAAtcaaccagcctggagg  Gtcgttcgcgcccagcaggtacaggcgcgccacggccgcgagaac  ttcggcgaacgcgaaagcgcctgtggggcggtcgttcgcgcccag  ctgctcgaccgacaactcgtgtgcgagcagttcggcgaacgcgaa  caggttctcactaccggcgccgcggccggcctgctcgaccgacaa  gtccttgtagtcgccctggaagtacaggttctcactacc  ATATATAAGCTTTCActtgtcgtcgtcgtccttgtagtcgcc |
| CB737 | *∆murA*::zeoR (converted to ∆*murA*::lox by vector pCreSacBR) | Tcgttgaactcatcgcaccacttctcca  TACATTATACGAAGTTATcacagcaacttcgcctgata  tatcaggcgaagttgctgtgATAACTTCGTATAATGTA  gtggaacacatcgtggacctATAACTTCGTATAGCAT  ATGCTATACGAAGTTATaggtccacgatgtgttccac  aacaacccatccaaagacaggtgaattc |
|  | pKK158 -P*_cwlM_*-*cwlM* | ATATATTCTAGA aacgcatcgtcggcgccaa  ATATATTTAATTAAaggctggttgacggcaat  ATATATAAGCTtcaggcgccgcggccggcctgct |
|  | pDE43MCtZ -P*_murA_*-*murA*-strep | GGGGACAACTTTGTATAGAAAAGTTGCCttccacagcacatcaccctc  GGGGACTGCTTTTTTGTACAAACTTGCtagacaggccgcagatcgga  GGGGACAAGTTTGTACAAAAAAGCAGGCTAGGAAGacctgcgtgagcgagcgtttc  CTTCTCGAACTGGGGGTGGCTCCAGTCcgagcttactctctcgatctc  GGGGACCACTTTGTACAAGAAAGCTGGGTGctaCTTCTCGAACTGGG |
| CB757 | pET28 – his-MurA_TB_ | gcctggtgccgcgcggcagccatatggccgagcgtttcgtcgtga  ggtgctcgagtgcggccgcaagcttctaacagcatacccgttcga |
| CB762 | pDE43MCtZ –P*murA*-*murA* S368P-strep | GGGGACAACTTTGTATAGAAAAGTTGCCttccacagcacatcaccctc  GGGGACTGCTTTTTTGTACAAACTTGCtagacaggccgcagatcgga  GGGGACAAGTTTGTACAAAAAAGCAGGCTAGGAAGacctgcgtgagcgagcgtttc  GGGGACCACTTTGTACAAGAAAGCTGGGTGctaCTTCTCGAACTGGG  Caccggcccggatgtccggcgaccacacgggtgc  gcacccgtgtggtcgccggacatccgggccggtg |
| CB706 | pKK216-*lprG*-strep-cys | AATTAAGAAGGAGATATACAT atgcggacccccagacgc  ACTTCTCGAACTGTGGGTGGCTCCAGTCgctcaccgggggcttcgt  CCAATTAATTAGCTAAAGCTTctaCTTCTCGAACTGGGGGTGGC |
| CB884 | pET28 – his-MurA_MS_ | gcctggtgccgcgcggcagccatatgagcgagcgtttcgtggtg  ggtgctcgagtgcggccgcaagctt ctacgagcttactctctc |
| CB885 | pET28 – his-MurA_MS_S368P | gcctggtgccgcgcggcagccatatgagcgagcgtttcgtggtg  ggtgctcgagtgcggccgcaagctt ctacgagcttactctctc  Caccggcccggatgtccggcgaccacacgggtgc  gcacccgtgtggtcgccggacatccgggccggtg |
| CB886 | pKK216 - pgm-strepC | TGCTTAATTAAGAAGGAGATATACATatgaggatccgtcggctggtga  CACTTCTCGAACTGGGGGTGGCTCCAGTCcttggcttcgagccgcgggaa  GGTCCCCAATTAATTAGCTAAAGCTTTCAACACTTCTCGAACTGGGG |
| CB899 | pHGWA – his-CwlM_TB_ T382A | Ggggacaagtttgtacaaaaaagcaggcttcatgccgagtccgcgccgcgaa  GGGGACCACTTTGTACAAGAAAGCTGGGTGtcaagaaccgccgagtctacccg  Gttaggcaagaacgatcggcccgccggcacattcactttcgccgag  ctcggcgaaagtgaatgtgccggcgggccgatcgttcttgcctaac |
| CB900 | pHGWA – his-CwlM_TB_ T382A T384A T386A | Ggggacaagtttgtacaaaaaagcaggcttcatgccgagtccgcgccgcgaa  GGGGACCACTTTGTACAAGAAAGCTGGGTGtcaagaaccgccgagtctacccg Tgttaggcaagaacgatcggcccgccggcgcattcgctttcgccgagttgctggcccacg  cgtgggccagcaactcggcgaaagcgaatgcgccggcgggccgatcgttcttgcctaaca |

| **Supplementary file 1D. Kinetic parameters of MurA proteins.** | | | | | |
| --- | --- | --- | --- | --- | --- |
| Species | Protein | UDP-GlcNAc Km, uM | PEP Km, uM | Kcat, s^-1^ | Reference |
| *E. coli* | MurA | 6.3 | 4.1 | 8.9 | (Dai et al., 2002) |
| *E. coli* | MurA | 15 | 0.4 | 3.8 | (Kim et al., 1996) |
| *E. cloacae* | MurA | 80 | 8.3 | 3 | (Krekel et al., 2000) |
| *S. aureus* | MurA | 124 | 11.5 | 1.1 | (Blake et al., 2009) |
| *S. aureus* | MurZ | 169 | 11.6 | 0.8 | (Blake et al., 2009) |
| *S. pneumoniae* | MurA1 | 244 | 37 | 0.41 | (Du et al., 2000) |
| *S. pneumoniae* | MurA2 | 119 | 11 | 0.78 | (Du et al., 2000) |
| *M. tuberculosis* | MurA | 2743 | 199 | 0.033 | (Xu et al., 2014) |
| *M. smegmatis* | MurA | 2320 | 121 | 0.147 | (Xu et al., 2014) |
